# Breed-Associated Differences in Differential Gene Expression Following Immunotherapy-Based Treatment of Canine High-Grade Glioma

**DOI:** 10.3390/ani15010028

**Published:** 2024-12-26

**Authors:** Susan A. Arnold, Walter C. Low, Grace Elizabeth Pluhar

**Affiliations:** 1 Department of Veterinary Clinical Sciences, University of Minnesota, Saint Paul, MN 55108, USA; pluha006@umn.edu; 2 Department of Neurosurgery, University of Minnesota, Minneapolis, MN 55455, USA; lowwalt@umn.edu; 3 Masonic Cancer Center, University of Minnesota, Minneapolis, MN 55455, USA

**Keywords:** canine glioma, high-grade glioma, immunotherapy, tumor transcriptomics

## Abstract

Canine high-grade glioma (HGG) is among the deadliest and most treatment-resistant forms of canine cancer. Successful, widespread treatment is challenged by heterogeneity in tumor cells and the tumor microenvironment and tumor evolution following treatment. Immunotherapy is theoretically a strong novel therapy, since HGG-generated immunosuppression is a substantial malignancy mechanism. Immunotherapy has improved survival times overall, but has been associated with extremely poor outcomes in French bulldogs. Given this breed-specific observation, we hypothesized that within the French bulldog breed, there are key transcriptomic differences when compared to other breeds, and that their tumors change differently in response to immunotherapy. Using bulk RNA sequencing, French bulldog tumors were confirmed to differ substantially from boxer and Boston terrier tumors, with only 15.9% overlap in significant differentially expressed genes (DEGs). In upregulated DEGs, the magnitude of changes in expression post-treatment compared to pre-treatment was markedly greater in French bulldogs. Gene set enrichment analysis confirmed that following treatment, French bulldog tumors showed enrichment of key immune-associated pathways previously correlated with poor prognosis. Overall, this study confirmed that French bulldog HGG transcriptomes differ from boxer and Boston terrier transcriptomes, further refining description of the canine glioma transcriptome and providing important information to guide novel therapy development, both for specific dog breeds and for possible correlative variants of human glioblastoma.

## Simple Summary

Canine high-grade glioma (HGG) is a deadly disease in dogs, with limited treatment options. Involving the host immune system in the fight against HGG through immunotherapy has improved outcomes in many dogs, with a notable exception in the French bulldog breed. We hypothesized that French bulldog HGG responds differently to immunotherapy than other breeds, and that this response difference is attributable to differences in transcriptomic landscapes in the breed compared to other breeds. We performed bulk RNA sequencing (RNASeq) on tumors pre-treatment and post-treatment and examined the differentially expressed genes (DEGs) between the two timepoints in both French bulldogs and in our comparison group of boxers and Boston terriers. We then analyzed the differences in DEGs between French bulldogs and boxers and Boston terriers, and performed gene set enrichment analysis (GSEA) to categorize the observed changes by gene function. We confirmed that when compared to boxers and Boston terriers, the transcriptomes of French bulldog tumors change in significantly different ways following immunotherapy treatment, with many DEGs mapping to tumor-promoting pathways and immune pathways. This study highlights important breed-associated differences in HGG transcriptomes, which may guide patient-centered immunotherapy treatment to improve patient outcomes.

## 1. Introduction

High-grade glioma (HGG), which develops spontaneously at similar rates in both people and pet dogs, is one of the most complex, treatment-resistant cancers [1]. In dogs, glioma represents up to 70% of primary brain tumors, affecting approximately 10 per 100,000 dogs [2,3]. The median age at diagnosis is eight years, and certain breeds are at a higher risk for developing HGG [3,4,5]. Brachycephalic breeds, specifically those belonging to the bulldog phylogenetic clade, develop glioma at higher frequencies than other dog breeds, accounting for over 50% of all glioma diagnoses, especially oligodendroglioma [5]. A genomic region on canine chromosome (CFA) 26 is strongly associated with glioma risk in dogs. This region is proposed to have been under selection in the creation of breeds belonging to the bulldog phylogenetic clade [6]. Although brachycephalic dogs represent most glioma-bearing patients, much of the research on canine glioma has not included brachycephalic breeds proportionate to the breed-associated glioma frequency [1,7,8]. Given that dog breeds such as boxers, Boston terriers, French bulldogs, and English bulldogs, represent the majority of dogs that develop glioma, clade- and breed-specific studies investigating glioma are needed to further our understanding of canine glioma [1,7].

In both people and dogs, there are limited treatment options for glioma [2,9]. Regardless of treatment, complete remission rates are extremely low [2,9]. Thus, there is an intense effort to develop novel therapies to improve outcomes. One of glioma’s most potent weapons is its hold over the immune system. Glioma is both locally and systemically immunosuppressive, and this feature substantially impacts treatment effectiveness [10]. Immunotherapy-based treatment is theoretically promising, as it involves augmenting or facilitating the host’s immune system to enable anti-tumor immune responses. Various immunotherapy approaches include immune checkpoint inhibitors (ICI), bi-specific antibodies, oncolytic viruses, cancer vaccines, adoptive cell therapy, and allogeneic cell therapy [11]. Many of these are under current investigation to treat HGG.

As pet dogs with HGG are a strong model for human HGG, particularly the common and deadliest form, glioblastoma (GB), they are ideal candidates for preclinical trials to study various novel therapies [1,8,12]. The goal of such trials is to not only provide a possible superior treatment option to pet dogs, for which there is currently no gold-standard HGG treatment, but also to provide meaningful clinical outcomes to guide novel therapy development for people with GB [12]. Thus, our group studies novel immunotherapy-based treatments for canine HGG.

The focus of our immunotherapy research has been on combining autologous tumor lysate (ATL) with an ICI for the CD200 immune checkpoint. CD200 is an immune checkpoint glycoprotein that is highly expressed in glioma [13]. By binding to CD200 inhibitory receptors (CD200R) on the cell membranes of immune cells, glioma-secreted CD200 suppresses a normal immune response to the tumor [13]. Thus, an immune checkpoint inhibitor (ICI) to block the immunosuppressive effect of CD200 was developed and used in conjunction with ATL therapy in the dogs presented herein, following surgical tumor resection [14]. The CD200 immune checkpoint interacts with many other signaling pathways, including the PD/PD-L1, PI3K-Akt, and Ras-MEK-ERK pathways, but many interactions are yet to be determined [14,15].

When this therapy protocol was implemented, a clear schism in patient outcomes was observed: while overall, the median survival time was 387 days, in treated French bulldogs, the median survival time was 48 days [16]. Histopathologically, all tumors were confirmed as high-grade glioma, and all dogs were similar in age and had similar distributions of tumor locations. Thus, given the current paucity of breed-specific data on canine HGG, and this marked survival disparity in French bulldogs compared to other breeds, we sought to characterize the differential gene expression of French bulldog tumors versus tumors of dogs from the same phylogenetic clade (boxers and Boston terriers) by examining how differential gene expression changes following immunotherapy in French bulldog HGG compared to boxer/Boston terrier HGG.

Examining how tumors respond to therapy is important, as it can explain clinical response, enable protocol adjustments, and provide insight into the complex tumor microenvironment (TME). Characterizing tumor response is especially important for novel therapy development, such as immunotherapy. There have been transcriptomic studies in human glioma examining differential gene expression following treatment, including in response to ICI [17,18,19,20]. These studies have illuminated how glioma changes over time differently in response to different treatment types, the role of the TME in tumor evolution, and in some cases, have identified specific DEGs that correlate with treatment response [20].

Thus, the purpose of this study was to characterize breed-associated changes in HGG transcriptomes post-treatment compared to pre-treatment. To achieve this goal, we performed bulk RNASeq on HGG samples obtained from French bulldogs, boxers, and Boston terriers. In paired analyses, we determined the differential gene expression profiles of French bulldogs post-treatment versus pre-treatment, and boxers and Boston terriers post-treatment versus pre-treatment. We then characterized the difference in log2-fold changes in DEGs between the breed groups and performed gene set enrichment analysis (GSEA) to reveal differences in enriched pathways between the breed groups. We confirmed that the tumors of French bulldogs undergo different transcriptomic changes following immunotherapy when compared to the transcriptomic changes in boxers and Boston terriers. In particular, GSEA demonstrated a higher proportion of immune-related enriched pathways in French bulldogs compared to boxers and Boston terriers.

Characterizing breed-associated differences enables refinement of the canine model and the ability to predict treatment response in each canine patient. The results of this study provide important breed-specific RNASeq data that can inform not only canine brain tumor treatment, but also human GB treatment.

## 2. Materials and Methods

### 2.1. Glioma Samples

High-grade glioma samples were obtained prior to immunotherapy and after immunotherapy as biopsy specimens submitted to the Comparative Pathology Shared Resource (CPSR) at the University of Minnesota. Sample collection ranged from 2005 to 2015. The samples used in this report were obtained as part of enrollment in clinical trials for pet dogs with HGG, approved by the University of Minnesota Institutional Animal Care and Use Committee (IACUC) under protocols 2001-37742A, 2002-37886A, 2111-39571A, and 2111-39569A. Patient breed, sex, age, tumor location, and overall survival (OS) were obtained from medical records. All French bulldogs, boxers, and Boston terriers for which there were both pre-treatment and post-treatment biopsy samples available were included in the study. All enrolled dogs were free of comorbidities, including endocrine diseases and other cancers.

Prior to immunotherapy, all dogs were treated with gross total surgical resection consisting of either a transfrontal or a modified rostrotentorial craniotomy, depending on the tumor location. Immediate post-operative magnetic resonance imaging (MRI) studies were performed and evaluated by board-certified veterinary radiologists to confirm gross total resection. Following resection, treatment consisted of ATL and CD200 ICI. One French bulldog received gene therapy consisting of delivery of the Flt3 ligand in combination with temozolomide, before undergoing a second surgical resection for tumor regrowth and subsequent treatment with ATL and CD200 ICI. Another French bulldog received temozolomide in addition to ATL and CD200 ICI.

Biopsy specimens (surgically resected tumor tissue for pre-treatment samples and whole brains for post-treatment samples) were fixed in 10% neutral buffered formalin, then embedded in paraffin wax. A board-certified veterinary pathologist reviewed all submissions and confirmed a diagnosis of high-grade glioma in all cases based on the criteria described by Koehler et al. [21]. To ensure that tumor tissue was present in the post-treatment samples, slides were examined using light microscopy. Sections with confirmed tumor tissue were selected. To prepare the samples for RNA sequencing, 6–10 μM scrolls were created.

### 2.2. RNA Purification and RNA Sequencing

The University of Minnesota Genomics Center (UMGC) performed RNA extraction and RNA sequencing. For extraction, the PureLink TM FFPE Total RNA Isolation kit (Invitrogen, Carlsbad, CA, USA) was used. Following RNA elution, DNAase I digestion was performed to provide DNA-free total RNA. A fluorimetric RiboGreen assay (Life Technologies, Carlsbad, CA, USA) was used to quantify total eukaryotic RNA isolates. Capillary electrophoresis on the Agilent BioAnalyzer 2100 (Agilent, Santa Clara, CA, USA) was used to assess total RNA integrity, generating an RNA integrity number (RIN). RIN scores above 3 were deemed sufficient for further processing. Those samples that passed quality control then underwent library preparation using the SMARTer Stranded Total RNA-Seq Kit v2, Pico Input Mammalian (Takara Bio, Mountain View, CA, USA). This kit removes ribosomal RNA. The NovaSeq 6100 S1 flow cell (Illumina, San Diego, CA, USA) was used to perform RNA sequencing at a targeted depth of ≥40 million reads per sample.

The Minnesota Supercomputing Institute (MSI) processed RNASeq data using PURR, a pipeline of the Collection of Hierarchical UMII/RIS Pipelines (CHURP). CHURP was developed by a group at the Minnesota Supercomputing Institute, and the analysis is provided as part of the RNASeq package run through the UMGC [22]. Two 50 bp FastQ paired end reads for 29 samples (*n* = 61.2 million average reads per sample) were trimmed using Trimmomatic (v 0.33) enabled with the optional “headcrop-3” option, “-q” option, and 3 bp sliding-window trimming from 3’ end requiring minimum Q30. Quality control on raw sequence data for each sample was performed with FastQC. Read mapping was performed via Hisat2 (v2.1.0) using the dog genome (Dog10K_Boxer_Tasha/canFam6 NCBI RefSeq assembly GCF_000002285.5) as reference [23]. Gene quantification was performed via Feature Counts for raw read counts.

### 2.3. Differential Gene Expression, Comparisons Between French Bulldogs and Boxers and Boston Terriers, and Gene Set Enrichment Analysis

Using DESeq2 in R, two differential gene expression analyses were performed. First, DEGs in French bulldogs were evaluated by comparing gene expression post-treatment to pre-treatment. Second, DEGs in boxers and Boston terriers were evaluated by comparing gene expression post-treatment to pre-treatment. Both analyses used paired samples, such that each dog was compared to itself within the analysis.

For each DESeq2 analysis, the data were visually inspected for any outliers. Normalization was performed using a median-of-ratios method. Principal component analyses were performed to determine the proportion of variance attributable to the sampling times of post-treatment versus pre-treatment. Raw counts for each gene were fitted to the DESeq2 negative binomial model. Dispersion was confirmed to decrease with increasing mean. Shrinkage was then applied to the data. First, MA plots were created to examine the mean of the normalized counts versus log2-fold changes for all genes tested. To improve estimated fold changes, log2-fold change shrinkage was applied. The shrunken data were then used to create subsequent MA plots to confirm improved differential expression accuracy.

Following shrinkage, significant DEGs were then extracted for further evaluation and visualization. The Wald test for differential expression was used to identify significant DEGs, using the Benjamini–Hochberg-adjusted *p*-value for multiple test correction. DEGs were considered significant if they had an adjusted *p*-value of <0.05. An absolute log2-fold change > 1, corresponding to an absolute fold change > 2, was used as a cutoff for identifying investigable DEGs. The numbers of significant DEGs were determined in both groups and were visualized for French bulldog DEGs post-treatment versus pre-treatment and boxer and Boston terrier DEGs post-treatment versus pre-treatment as heatmaps. Then, to further examine differences in expression between the breed groups, the significant DEGs of each group were investigated in the other group and visualized as heatmaps.

To compare the log2-fold changes that occurred, the French bulldog and boxer and Boston terrier data sets were combined into a single data frame. A Wilcoxon rank-sum test was used to determine whether there were significant differences in DEGs between French bulldogs and boxers and Boston terriers. A Jaccard index calculation was performed to quantify how many overall DEGs and significant DEGs were shared between French bulldogs and boxers and Boston terriers. The log2-fold changes in all DEGs were visualized in French bulldogs relative to boxers and Boston terriers.

Given the small sample size, permutation testing was also performed within each DESeq2 analysis to investigate the intragroup consistency of identified DEGs. To capture the total of five French bulldogs in all possible groups of two, a total of 10 permutations were included. To capture the total of four boxers and Boston terriers in all possible groups of two, a total of 6 permutations were included. Results were compiled to record the frequency that each gene was identified as a DEG.

Four DEGs groupings were created: the top 25 DEGs that increased the most in post-treatment French bulldogs, the top 25 DEGs that decreased the most in post-treatment French bulldogs, the top 25 DEGs that increased the most in post-treatment boxers and Boston terriers, and the top 25 DEGs that decreased the most in post-treatment boxers and Boston terriers. In each of these analyses, included DEGs were those that were significant with an absolute log2-fold change > 1 in the breed group of interest.

The differences in log2-fold changes were calculated by subtracting the log2-fold changes of boxers and Boston terriers from the log2-fold changes of French bulldogs. The DEGs included in this analysis were limited to DEGs that were significant in French bulldogs with an absolute log2-fold change > 1. Subtraction rather than division was selected as this is the preferred method for comparing log2-fold changes between two groups [24]. Since the data are already log2 transformed, the calculated difference is equivalent to calculating the ratio between original expression levels [24]. An unpaired *t*-test was performed using the Benjamini–Hochberg-adjusted *p*-value for multiple test correction. Significance was set at adjusted *p*-value < 0.05.

DEGs were sorted by the degree of difference in log2-fold changes between breed groups. The top 50 DEGs with the largest absolute change were extracted for further evaluation. The differences in log2-fold changes between French bulldogs and boxers and Boston terriers were visualized. DEGs whose expression direction differed between French bulldogs and boxers and Boston terriers (for example, DEGs that were overexpressed in French bulldogs post-treatment compared to pre-treatment while being underexpressed in boxers and Boston terriers post-treatment compared to pre-treatment) were examined.

GSEA was performed for each paired comparison (French bulldogs post-treatment vs. pre-treatment and boxers and Boston terriers post-treatment vs. pre-treatment) to investigate enriched pathways. The R package “clusterProfiler” was used. Gene expression data were ranked by their differential expression in log2-fold change values. Positive values indicated upregulation and negative values indicated downregulation. Using predefined gene sets (GO and KEGG), enrichment scores were calculated based on the distribution of genes in the data compared to the predefined gene sets. Adjusted *p*-values were calculated using the Benjamini–Hochberg procedure to account for multiple testing. Dot plots were created to facilitate visualization of the different enriched pathways in French bulldogs versus boxers and Boston terriers.

## 3. Results

After searching through patient sample repositories, we identified five French bulldogs, two boxers, and two Boston terriers that had both pre- and post-treatment samples available for paired differential expression analysis. Within the French bulldog group, there were two neutered males and three spayed females. The mean age was six years (range 3 to 9.5 years). Within the boxer and Boston terrier group, there were two neutered male boxers, and two neutered male Boston terriers. The mean age was 6.8 years (range 4 to 9.5 years). Regarding treatment, following surgical resection, all dogs received autologous tumor lysate vaccination with our CD200 immune checkpoint inhibitor as intradermal injections. In addition to this therapy, one French bulldog also received temozolomide and another received temozolomide plus gene therapy initially but was treated with autologous tumor lysate vaccination and CD200 immune checkpoint inhibition following surgical resection of tumor regrowth.

**Figure 1:**
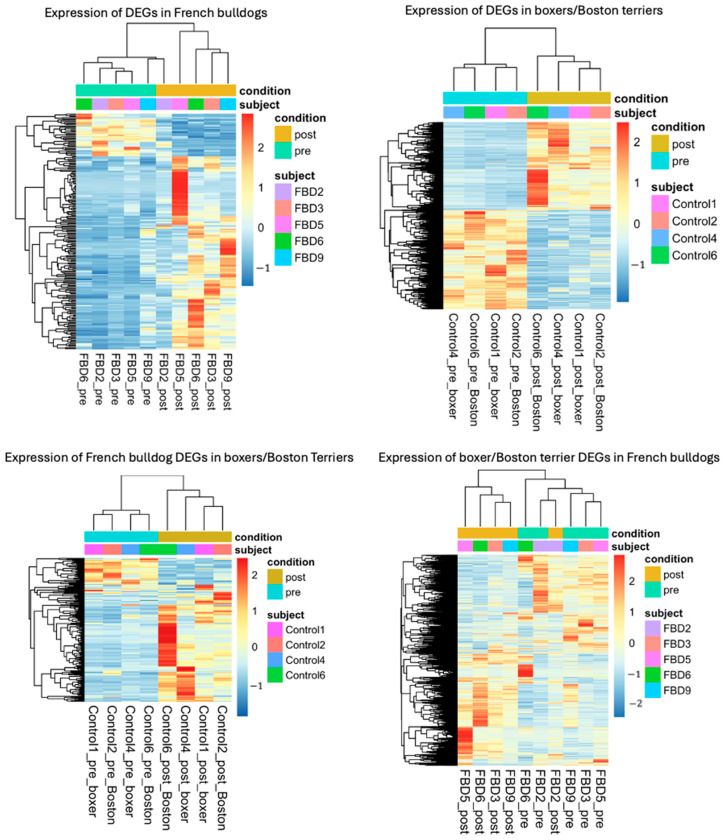
**Top left:** Heatmap showing significant DEGs with absolute log2-fold changes > 1 in French bulldogs; **Top right:** heatmap showing significant DEGs with absolute log2-fold changes > 1 in boxers and Boston terriers; **Bottom left:** heatmap showing expression patterns of the significant DEGs of French bulldogs in boxers and Boston terriers; **Bottom right:** heatmap showing expression patterns of the significant DEGs of boxers and Boston terriers in French bulldogs.

**Figure 2:**
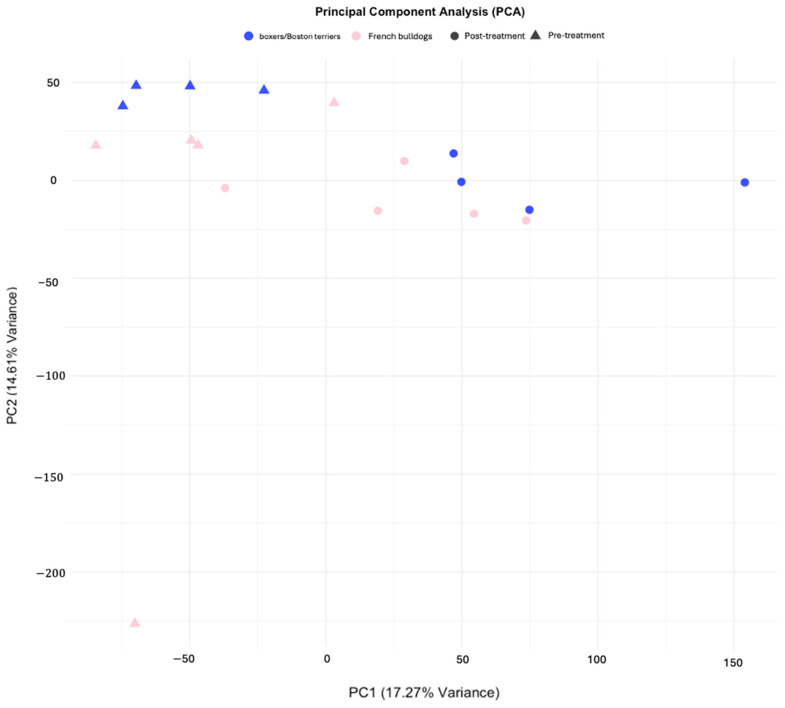
Principal component analysis showing merged RNASeq analyses of French bulldogs and boxers/Boston terriers. Samples clustered by treatment timepoint and breed.

In French bulldogs, there were 488 significantly upregulated DEGs and 269 significantly downregulated DEGs with absolute log2-fold changes > 1. In boxers and Boston terriers, there were 1749 significantly upregulated DEGs and 1924 significantly downregulated DEGs with absolute log2-fold changes > 1 (Figure 1). In both breed groups, samples arranged by timepoint (pre- or post-treatment) and display visible differences in expression patterns by timepoint. Investigation of significant DEGs of French bulldogs in boxers and Boston terriers, and vice versa, confirmed that the expression patterns differed between the breeds (Figure 1). A principal component analysis revealed that samples clustered strongly by treatment timepoint, but also moderately clustered by breed (Figure 2).

**Figure 3:**
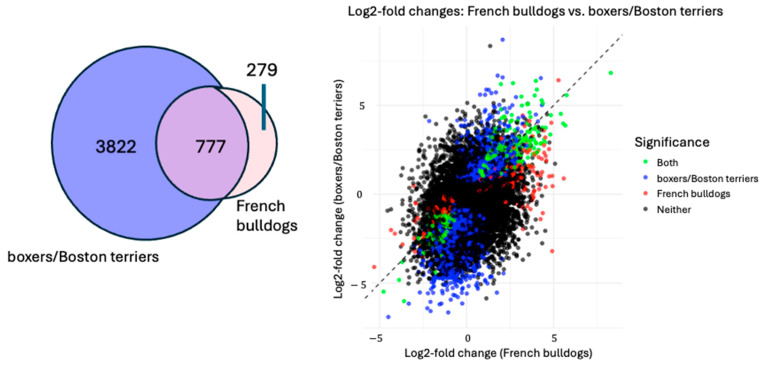
Visualization of DEGs in French bulldogs compared to Boxers and Boston Terriers. (**Left**): Venn Diagram showing DEGs exclusive to either breed group or shared between groups; (**Right**): scatterplot of the log2-fold changes in French bulldog DEGs versus boxer/Boston terrier DEGs.

When comparing the changes in differential gene expression post-treatment compared to pre-treatment for French bulldogs versus boxers and Boston terriers, the Wilcoxon rank-sum test showed that there was a significant difference in expression between the two groups (W = 438084303, *p* < 2.2 × 10^−16^). The Jaccard index was calculated as 0.159, indicating that there was only a 15.9% overlap in the significant DEGs between the two groups (Figure 3). When the log2-fold changes in DEGs were compared between French bulldogs and boxers and Boston terriers, French bulldogs overall had larger log2-fold changes than boxers and Boston terriers in the DEGs that were significant only in their respective groups, especially for upregulated DEGs (Figure 3).

**Figure 4:**
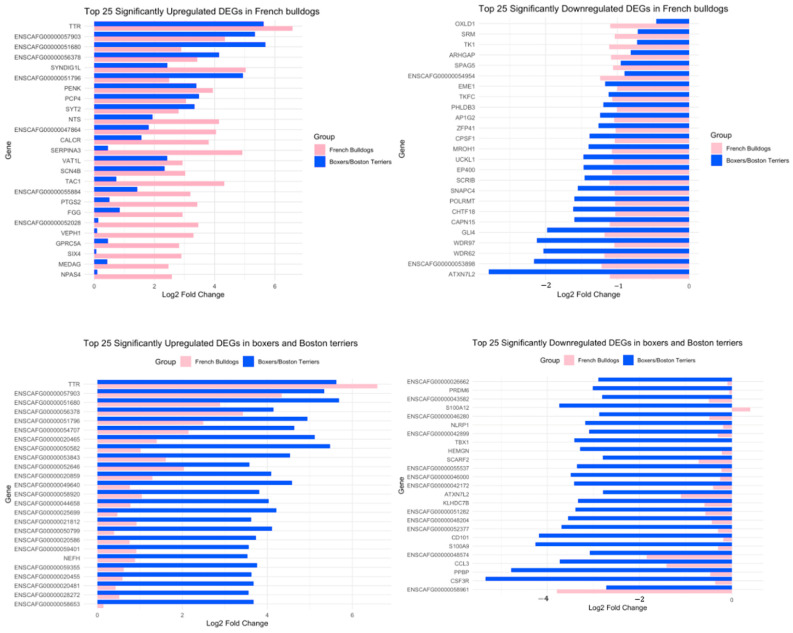
The top 25 upregulated and downregulated DEGs by breed group. For each analysis, the displayed DEGs were significant with an absolute log2-fold change > 1 in the breed group of interest. The expression of the alternate breed group for each DEG is provided for comparison. **Top left**: Top significantly upregulated DEGs in French bulldogs; **Top right**: top significantly downregulated DEGs in French bulldogs; **Bottom left**: top significantly upregulated DEGs in boxers and Boston terriers; **Bottom right**: top significantly downregulated DEGs in boxers and Boston terriers.

**Figure 5:**
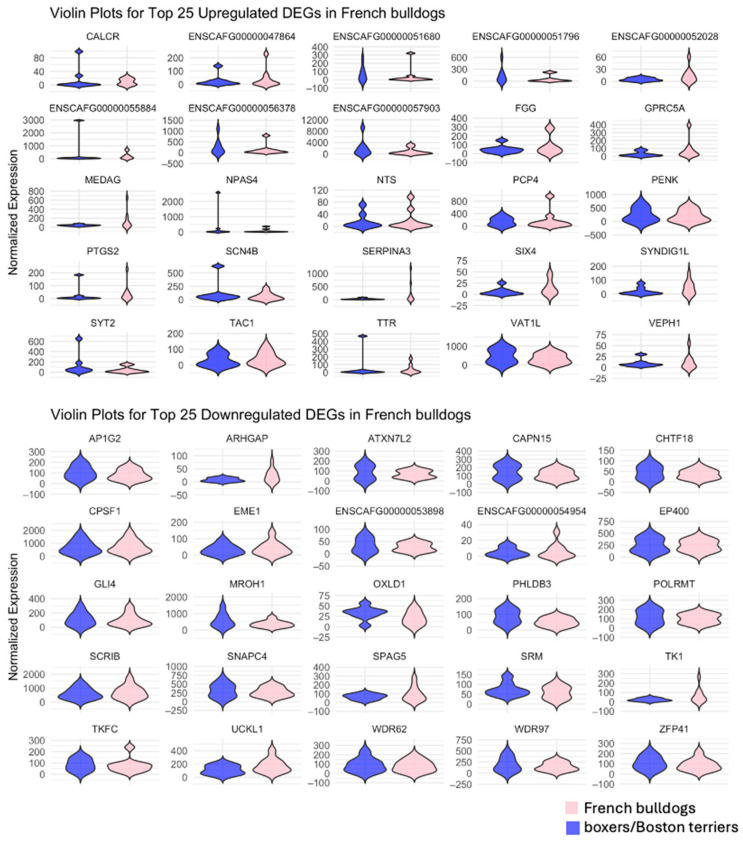
Violin plots for the top 25 upregulated (**Top**) and top 25 downregulated (**Bottom**) DEGs in French bulldogs.

**Figure 6:**
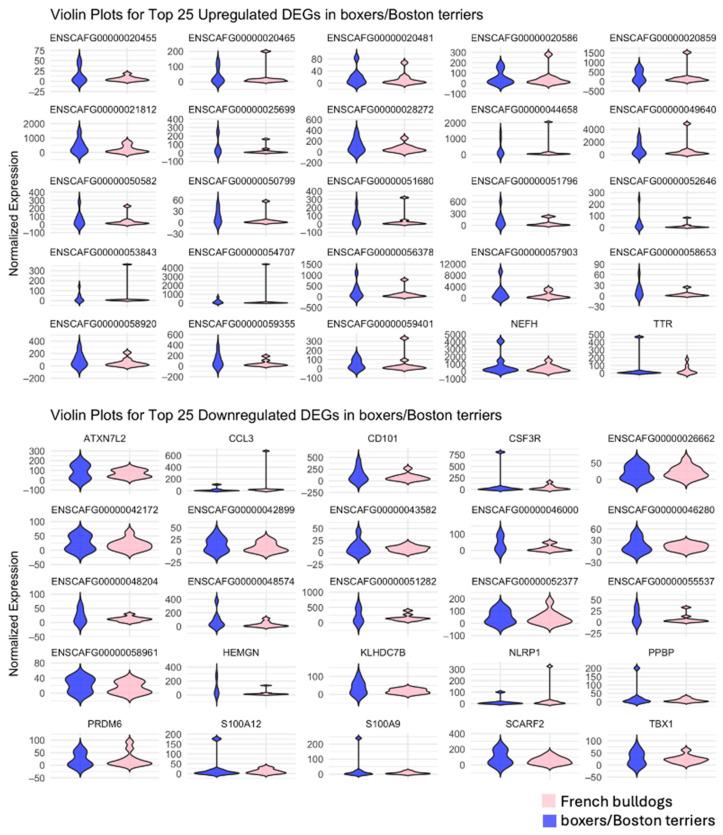
Violin plots for the top 25 upregulated (**Top**) and top 25 downregulated (**Bottom**) DEGs in boxers and Boston terriers.

Analysis of the top 25 significantly upregulated DEGs in French bulldogs, top 25 significantly downregulated DEGs in French bulldogs, top 25 significantly upregulated DEGs in boxers and Boston terriers, and top 25 significantly downregulated DEGs in boxers and Boston terriers showed that there was little overlap in the DEGs between the breed group of interest and the comparison breed group (Figure 4). In general, upregulated DEGs in French bulldogs had higher magnitudes of log2-fold changes compared to boxers and Boston terriers, and downregulated DEGs in French bulldogs had lower magnitudes of log2-fold changes compared to boxers and Boston terriers (Figure 4, Figure 5 and Figure 6). Conversely, for the top downregulated DEGs in boxers and Boston terriers, corresponding changes in log2 fold expression in French bulldogs had lower magnitudes (Figure 4, Figure 5 and Figure 6).

**Figure 7:**
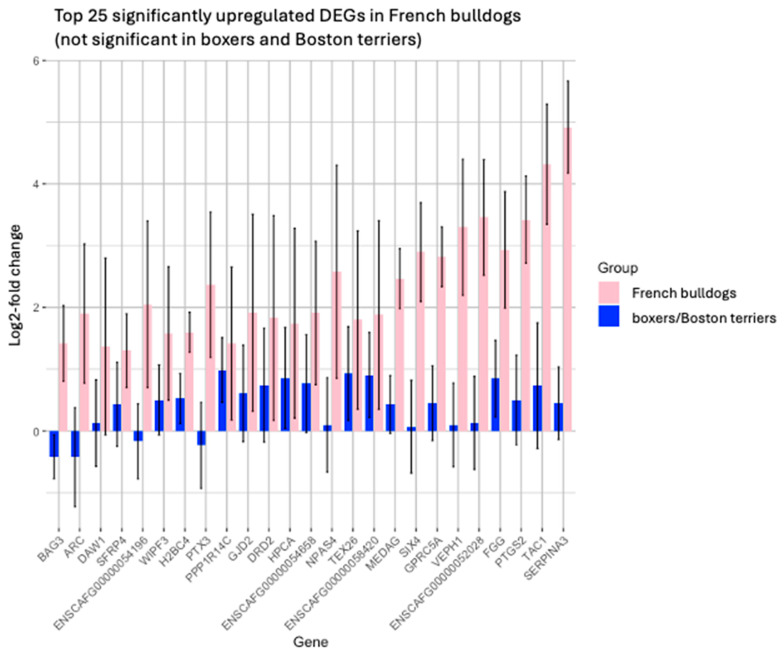
Barplot with error bars of the top 25 significantly upregulated DEGs in French bulldogs that were not significant in boxers and Boston terriers.

Given the observation that upregulated DEGs that were significant only in French bulldogs had overall larger log2-fold changes post-treatment compared to pre-treatment, we then isolated the top 25 upregulated DEGs that were significant with a log2-fold change > 1 in French bulldogs that were not significant in boxers and Boston terriers to correspond to the data visualized in Figure 3 (Figure 7). Like the other data sets, many of these DEGs were non-coding DEGs. Protein-coding upregulated DEGs in French bulldogs that had a high magnitude of log2-fold change following treatment included TAC1, PTGS2, FGG, VEPH1, GPRC5A, SIX4, MEDAG, TEX26, NPAS4, HPCA, DRD2, GJD2, PTX3, WIPF3, SFRP4, DAW1, and BAG3. There is overlap in error bars for some of the DEGs, likely due to larger variability associated with our small sample size. Nonetheless, these differences in expression were extensive between breeds and thus were considered noteworthy.

**Figure 8:**
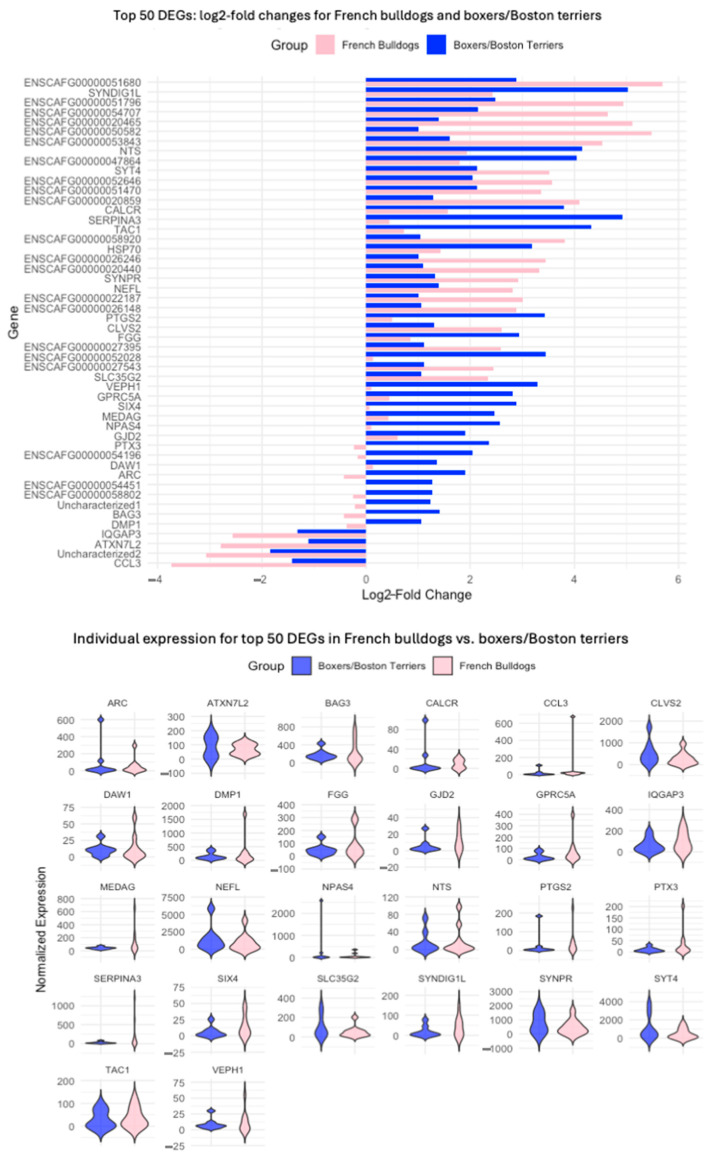
The top 50 DEGs with the largest difference in expression following treatment between French bulldogs and boxers/Boston terriers represented as a bar plot (**Top**) and violin plots (**Bottom**). Note that for some DEGs, the direction of log2-fold change post-treatment compared to pre-treatment was opposite in French bulldogs versus boxers and Boston terriers.

To examine the significance of the difference between log2-fold changes in French bulldogs and boxers and Boston terriers, we performed an unpaired *t*-test that demonstrated that the differences in log2-fold change expression were significant (padj = 7.59 × 10^−52^). We identified the top 50 genes with the largest differences in absolute log2-fold changes between French bulldogs and boxers and Boston terriers by subtracting the log2-fold changes in boxer/Boston terrier DEGs from those of French bulldogs (Figure 8). In 14 DEGs, the absolute difference in log2-fold changes exceeded 2.5, meaning that expression of these DEGs was 2.5 times higher in French bulldogs than in boxers and Boston terriers. In seven of the significant DEGs in French bulldogs with an absolute log2-fold change > 1, the direction of log2-fold change was inverted in boxers and Boston terriers. None of these DEGs were significant in boxers and Boston terriers.

**Figure 9:**
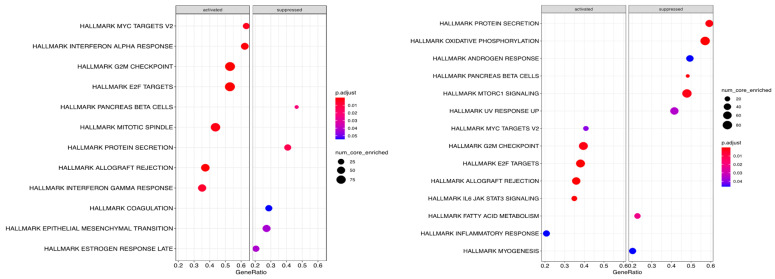
Gene set enrichment analyses. (**Left**): In French bulldogs; (**Right**): In boxers and Boston terriers.

GSEA of French bulldogs showed that post-treatment, twelve pathways were significantly enriched, consisting of seven activated and five suppressed pathways (Figure 9. GSEA of boxers and Boston terriers showed that post-treatment, fourteen pathways were significantly enriched, consisting of six activated and eight suppressed pathways (Figure 9).

Among the enriched pathways, French bulldogs and boxers and Boston terriers shared enriched activated pathways of myc targets, G2M checkpoint, and E2F targets, and suppressed activated pathways of protein secretion and pancreas beta cells. Activated pathways in French bulldogs that were not observed in boxers and Boston terriers included interferon alpha response, interferon gamma response, and mitotic spindle. Suppressed pathways in French bulldogs that were not observed in boxers and Boston terriers included coagulation, epithelial-to-mesenchymal transition, and late estrogen response. Activated pathways in boxers and Boston terriers that were not observed in French bulldogs included JAK STAT3 signaling and inflammatory response. Suppressed pathways in boxers and Boston terriers that were not observed in French bulldogs included oxidative phosphorylation, androgen response, MTORC1 signaling, UV response, fatty acid metabolism, and myogenesis.

## 4. Discussion

This study provides a comprehensive evaluation of differential gene expression in two breed groups of glioma-bearing dogs treated with vaccine and immune checkpoint inhibitor-based immunotherapy. There were several purposes for conducting this study. First, the impetus for this study was to elucidate the reason for a disparity in treatment response in French bulldogs compared to other breeds when treated with ATL and CD200 ICI by determining if there were differences in expression patterns post-treatment compared to pre-treatment. Subsequently, target genes could be identified as drivers of breed-specific tumor phenotypes, which could enable treatment tailoring by breed. Finally, given the robustness of the canine glioma model for human glioblastoma (GB), we sought to add transcriptomic information to the growing database of canine glioma information [1,5,25,26,27]. To date, there are no studies that disclose differences in response to treatment by breed. Such information is not only valuable when providing treatments for these dogs, but also in continuing the refinement of the canine glioma model and in considering trial design and the impacts of breed inclusion on trial results, which could drastically alter whether a given treatment is deemed eligible for exploration in human treatment.

All the dogs in this study were treated with a combination of autologous tumor lysate (ATL) and a peptide ligand that interferes with the CD200 immune checkpoint, yet there was a marked disparity in survival time between French bulldogs and boxers and Boston terriers. All dogs were confirmed to have gross total resection. Between pre-treatment and post-treatment sampling, one French bulldog previously had undergone an initial surgical resection followed by treatment with Flt3 ligand gene therapy and temozolomide, and another French bulldog received temozolomide in addition to ATL and CD200 ICI. These dogs were included because assessment of their DEGs post-treatment versus pre-treatment did not differ substantially from other French bulldogs. This suggests that the additional treatments did not have critical impacts on the genetic evolution of their tumors and TME. Overall, this is a small data set with low power, intended to provide compelling preliminary evidence for breed-associated differences in anti-glioma immunotherapy response.

To investigate the differences in differential gene expression between these breeds, we started by performing paired differential gene expression analyses on French bulldogs post-treatment versus pre-treatment and did the same for boxers and Boston terriers. We paired the samples such that each pre-treatment sample was matched in the analysis with the post-treatment sample corresponding to the same dog. Although this approach limited our sample size over performing this same analysis with dogs for which either a pre-treatment or post-treatment sample was not available, we chose to perform a paired analysis to eliminate confounding variables, reduce noise, improve power, and potentially contextualize group differences. The individual differential gene expression analyses demonstrated that samples clustered together by treatment timepoint in both French bulldogs and boxers and Boston terriers. This suggests that gene expression changed in association with treatment in both French bulldogs and boxers/Boston terriers.

We then sought to determine if gene expression changed differently in the two breed groups. First, we determined the significant DEGs in both breed groupings. Then, we examined the significant DEGs of each breed group in the other breed group, and confirmed via heatmap visualization that gene expression changed differently in French bulldogs compared to boxers and Boston terriers. We also examined merged, significant DEGs in French bulldogs and boxers and Boston terriers to examine how breed grouping impacted expression patterns. Although the most prominent clustering feature remained treatment timepoint, within each timepoint, most of the samples clustered by breed grouping, especially in the post-treatment cluster. This suggested that gene expression changes following treatment differed between French bulldogs and boxers and Boston terriers.

To determine the extent of difference in gene expression patterns between the breed groupings, we performed a Jaccard index analysis. This is a measure of the similarity between two sets of DEGs. This value ranges from 0 to 1 and is calculated as the intersection of the DEG sets divided by the union of the sets. A value of 0 means there is no overlap between the two sets, and a value of 1 means there is complete overlap. The Jaccard index for French bulldog versus boxer and Boston terrier DEG sets was 0.159, indicating that only 15.9% of the significant DEGs were significant in both breed groupings.

There were 279 significant DEGs that were unique to French bulldogs, compared to 3822 DEGs that were unique to boxers and Boston terriers. Among the top 25 significant upregulated and downregulated DEGs for each breed grouping, there was little overlap between French bulldogs and Boston terriers. Further, when evaluating the top 25 significantly upregulated DEGs in French bulldogs that were simultaneously not significantly upregulated in boxers and Boston terriers, among the protein-coding DEGs (TAC1, PTGS2, FGG, VEPH1, GPRC5A, SIX4, MEDAG, TEX26, NPAS4, HPCA, DRD2, GJD2, PTX3, WIPF3, SFRP4, DAW1, and BAG3), many of these DEGs have known roles in glioma. For example, PTGS2, an enzyme involved in prostaglandin synthesis, has been implicated in radiation resistance in human glioma [28]. GPRC5A, a G-protein-coupled receptor gene, is upregulated in many cancer types, including glioma [29,30,31,32]. SIX4 may promote angiogenesis in glioblastoma multiforme [33]. Overall, these findings demonstrate that, concerning DEGs in which expression differed the most between treatment timepoints, the way that tumors evolved over time and potentially in response to treatment differed substantially between French bulldogs and boxers and Boston terriers.

Further visualization of all identified DEGs demonstrated that the magnitude of log2-fold changes was overall larger in French bulldogs compared to boxers and Boston terriers, especially for upregulated DEGs. This suggested that, among the 279 DEGs that were significant exclusively in French bulldogs, those with the largest log2-fold changes may be responsible for the disparity in survival time observed in immunotherapy-treated French bulldogs. Whether this is due to French bulldog glioma being more prone to genetic evolution inherently or due to a breed-specific genomic response to immunotherapy is unknown. All dogs included in this study underwent treatment. If it had been ethically possible to include tumor samples from untreated dogs, resampled at later time points to provide “early” and “late” groupings to correspond with our pre- and post-treatment groupings, then this inclusion could help to determine the underlying cause of the larger magnitude of log2-fold changes observed in French bulldogs.

One of glioma’s mechanisms of malignancy is its ability to evolve over time [34]. Glioma cells have high plasticity, which allows them to adapt to stressors [35,36,37,38]. Gliomas evolve in several different ways, including branching and linear evolutionary patterns and therapy-related mesenchymal transitions [34,38,39,40,41,42,43]. Glioma co-evolves with the tumor microenvironment (TME), which is a complex, dynamic population of tumor-associated myeloid cells, dendritic cells, neutrophils, lymphocytes, blood–brain barrier constituents, astrocytes, and neurons, among others [44]. In comparison to treatment-naïve tumors, transcriptome analysis of bulk recurrent human glioblastoma samples following standard treatment (resection, radiation therapy, and temozolomide) demonstrated that hallmark glioma genes are not significantly altered. Rather, cell type diversity increases, corresponding with differential gene expression associated with a strong proportional increase in tumor-associated macrophages (TAMs) [45]. TAMs are key players in gliomagenesis and therapeutic resistance, and can drive transition to mesenchymal-like states [19,45,46].

As opposed to the upregulated DEGs, in which French bulldog DEGs had overall larger magnitudes of log2-fold changes post-treatment compared to pre-treatment, downregulated DEGs in French bulldogs exhibited relatively smaller changes in log2-fold change expression compared to boxers and Boston terriers. In fact, the magnitude of log2-fold change in downregulated DEGs in boxers and Boston terriers exceeded the magnitude of these changes in French bulldogs. The increased magnitude of downregulation in boxers and Boston terriers, which had superior clinical outcomes compared to French bulldogs, suggests that these downregulated DEGs may correspond to genes directly targeted by ATL and CD200 ICI therapy.

Alternatively, there were some important DEGs that were more downregulated in French bulldogs than in boxers and Boston terriers following treatment. The DEGs that experienced a greater degree of downregulation post-treatment in French bulldogs compared to boxers and Boston terriers include important immune constituents that improve immunotherapy effectiveness. For example, the chemokine CCL3 was more downregulated in French bulldogs post-treatment compared to boxers and Boston terriers (Figure 6). CCL3 is a potent innate and adaptive immune response activator [47]. Pro-inflammatory M1 macrophages secrete CCL3, and higher M1 CCL3 expression has been correlated with better survival in human glioma [48,49]. Thus, the French bulldog-specific downregulation of CCL3 may represent a key target for immunotherapy development specific to French bulldogs. Contrastingly, chronic stress leads to reduced CCL3 secretion, which results in reduced M1 macrophage infiltration and the exacerbation of an immunosuppressive TME [50]. Brain microvessel endothelial cells also secrete CCL3, regulating inflammatory responses across the blood–brain barrier. By augmenting T-cell reactivity, it promotes a robust immune response induction in response to immunotherapy [47]. That CCL3 expression decreased more in French bulldogs post-treatment compared to pre-treatment suggests that the immunotherapy treatment had a less robust immune response induction platform and a more immunosuppressive landscape on which to act, which may contribute to the poor treatment response in the breed.

To further characterize the expression profiles of French bulldog tumors compared to boxer and Boston terrier tumors, we performed GSEA. The GSEAs of French bulldogs compared to boxers and Boston terriers showed that, although they shared some similarities in enriched activated and suppressed pathways, there were some key differences. First, boxers and Boston terriers had more suppressed pathways than French bulldogs. Among the suppressed pathways in boxers and Boston terriers, MTORC1 signaling was significantly enriched, with many genes mapping to the pathway. Mammalian target of rapamycin (mTOR) mediates phosphatidyl-inositol-3-kinase (PI3L) signaling, the constitutive activation of which is a hallmark of glioblastoma [51]. Various mTOR inhibitors have been investigated as anti-glioma therapies [52]. Here, it is possible that the glioma of boxers and Boston terriers responded to ATL and CD200 ICI, potentially mediated by treatment-mediated suppression of the mTOR-PI3K axis. Alternatively, this observation is independent of the provided treatment. The relationship between the mTOR-PI3K axis and the CD200 immune checkpoint has not been elucidated. The GSEA results here suggest that examining this potential relationship could reveal a mechanism by which some canine glioma respond to ATL and CD200 ICI.

Another important difference in the GSEAs of French bulldogs compared to boxers and Boston terriers is that several immune-associated pathways of French bulldogs were activated, including interferon alpha response and interferon gamma response. This finding supports our consideration that differences in immune response, to either tumor cells, immunotherapy, or both, comprise an important constituent of the differences in gene expression in French bulldogs compared to boxers and Boston terriers.

## 5. Conclusions

In summary, this study confirms several important points. First, given the heterogeneity of canine glioma, and the genetic pools of purebred dog breeds, breed is an important variable in the transcriptomic profile of canine HGG, at least pertaining to the breeds studied herein [53]. Second, French bulldog glioma displays marked differences in gene expression following immunotherapy treatment compared to boxers and Boston terriers, especially in pathways attributable to the TME and immune system. Having identified key pathways, future studies to validate the findings are warranted, including quantitative real-time PCR, studying larger cohorts and additional dog breeds, and spatial transcriptomics to geographically contextualize gene expression. The results of this study provide breed-specific descriptions of HGG transcriptomes that augment our understanding of canine HGG and can heighten our understanding of the complex interplay between the tumors, the TME, and novel therapies.

## Data Availability

Data supporting reported results are openly available and can be found in the Data Repository for the University of Minnesota (DRUM): https://hdl.handle.net/11299/267779 (accessed on 15 November 2024).
