# Correction: Arnold et al. Breed-Associated Differences in Differential Gene Expression Following Immunotherapy-Based Treatment of Canine High-Grade Glioma. *Animals* 2025, *15*, 28

**DOI:** 10.3390/ani15202934

**Published:** 2025-10-10

**Authors:** Susan A. Arnold, Walter C. Low, Grace Elizabeth Pluhar

**Affiliations:** 1 Department of Veterinary Clinical Sciences, University of Minnesota, Saint Paul, MN 55108, USA; pluha006@umn.edu; 2 Department of Neurosurgery, University of Minnesota, Minneapolis, MN 55455, USA; lowwalt@umn.edu; 3 Masonic Cancer Center, University of Minnesota, Minneapolis, MN 55455, USA

**Text Correction**

There was an error in the original publication [1]. In the Discussion, the allograft rejection pathway was erroneously listed as upregulated in French bulldogs compared to boxers and Boston terriers.

A correction has been made to Results: Last paragraph should read as follows:

Among the enriched pathways, French bulldogs and boxers and Boston terriers shared enriched activated pathways of myc targets, G2M checkpoint, and E2F targets, and suppressed activated pathways of protein secretion and pancreas beta cells.

A correction has been made to Discussion:

Paragraph 12 should read as follows: Another important difference in the GSEAs of French bulldogs compared to boxers and Boston terriers is that several immune-associated pathways of French bulldogs were activated, including interferon alpha response and interferon gamma response. This finding supports our consideration that differences in immune response, to either tumor cells, immunotherapy, or both, comprise an important constituent of the differences in gene expression in French bulldogs compared to boxers and Boston terriers. 

Paragraph 13, which is below, should be removed:

The allograft rejection (AR) pathway has been studied in correlation with treatment responsiveness in human glioma patients [53,54]. Substantial infiltration of CD8 + T-cells and M1 and M2 macrophages was observed in high-AR but not in low-AR samples [53]. Further, the high-AR tumors were observed to have upregulation of many immune checkpoints, which could enable the tumors to evade the immune response more than in low-AR counterparts [53]. Finally, AR has been observed to be highly associated with epithelial growth factor receptor (EGFR) amplification, and is considered one of the most important pathways involved in observed immune responses in GBM [54,55]. Overall, the enrichment of the AR pathway in French bulldogs, but not in boxers and Boston terriers, marks a critically important breed-associated distinction that supports our hypothesis that the TME of French bulldog tumors differs from the TME of boxers and Boston terriers, and contributes to the disparity in survival outcomes. 

**References**

References 53–55 should be removed from the Conclusion, as the corresponding paragraph has been deleted. With this correction, the order of some references should be adjusted accordingly. 

The authors state that the scientific conclusions are unaffected. This correction was approved by the Academic Editor. The original publication has also been updated.
